# European hedgehogs (*Erinaceus europaeus*) as a natural reservoir of methicillin-resistant *Staphylococcus aureus* carrying *mecC* in Denmark

**DOI:** 10.1371/journal.pone.0222031

**Published:** 2019-09-06

**Authors:** Sophie Lund Rasmussen, Jesper Larsen, Rien E. van Wijk, Owen R. Jones, Thomas Bjørneboe Berg, Øystein Angen, Anders Rhod Larsen

**Affiliations:** 1 Department of Biology, University of Southern Denmark, Odense, Denmark; 2 Statens Serum Institut, Copenhagen, Denmark; 3 Van Wijk Eco Research, Søborg, Denmark; 4 Interdisciplinary Centre on Population Dynamics (CPop), Department of Biology, University of Southern Denmark, Odense, Denmark; 5 Naturama, Svendborg, Denmark; Instituto de Technologia Quimica e Biologica, PORTUGAL

## Abstract

**Objectives:**

A recent study from Sweden showed that European hedgehogs may constitute a reservoir for methicillin-resistant *Staphylococcus aureus* (MRSA), but this host-parasite relationship remains to be investigated in other countries. In this study, we therefore sought to: 1) determine the dissemination of MRSA in European hedgehogs throughout Denmark; 2) investigate determinants of MRSA carriage in hedgehogs; 3) determine the potential for zoonotic transmission of MRSA from hedgehogs to humans; and 4) characterise the detected MRSA on both a phenotypic and molecular level.

**Methods:**

Nasal swabs were taken from 188 dead hedgehogs collected by volunteers throughout Denmark to determine the occurrence of MRSA. Additionally, 16 hedgehog rehabilitators were tested for potential zoonotic transmission of MRSA from hedgehogs to humans. The swabs were incubated in tryptic soy broth supplemented with 6.5% NaCl, followed by spread of 10 μl on Brilliance MRSA 2 agar. One presumptive MRSA colony from each plate was subcultured on 5% blood agar. All *S*. *aureus* subcultures were verified by a PCR assay detecting *mecA*, *mecC*, *lukF-PV*, *scn*, and *spa*, followed by *spa* typing.

**Results:**

A total of 114 (61%) hedgehogs carried *mecC*-MRSA, whereas none carried *mecA*-MRSA. The detected *mecC*-MRSA belonged to two genetic lineages CC130 (*spa*-types: t528, t843, t1048, t3256, t3570, t6220, t17133) and CC1943 (*spa*-types: t978, t2345, t3391, t8835, t16868), 52% of which were *spa*-type t843 (CC130).The detection rate of *mecC*-MRSA in the hedgehogs was similar regardless of cause of death, sex, region and habitat type. None of the hedgehog rehabilitators carried MRSA.

**Conclusions:**

This nationwide study confirms a high occurrence of *mecC*-MRSA in hedgehogs, which could serve as a natural reservoir for this specific type of MRSA. Furthermore, our study did not find signs of zoonotic transmission of *mecC*-MRSA to hedgehog rehabilitators.

## Introduction

*Staphylococcus aureus* is a commensal bacterium with the potential to cause severe infections in humans. Methicillin-resistant *S*. *aureus* (MRSA) are resistant to most β-lactam antibiotics, due to the expression of additional penicillin binding proteins (PBPs) with low affinity to β-lactams, which are encoded by either *mecA*, *mecB* or *mecC* genes, of which *mecA* and *mecC* are carried in mobile genetic elements, referred to as staphylococcal cassette chromosome *mec* (SCC*mec*) [1, 2]. MRSA is a major cause of hospital-associated infections in Europe [3], and since the 1990s there has been a rise in community-associated MRSA infections among people with no apparent risk factors for contracting MRSA [4]. During the last decade, livestock-associated MRSA has additionally been recognized as a third major cause of MRSA infections in humans [3, 5, 6].

The *mecA* gene predominates in human MRSA isolates, and it was not until 2011 that the *mecC* gene was described in *S*. *aureus* from humans and dairy cattle from Denmark, England, Ireland and Scotland [7, 8]. Subsequently, *mecC*-MRSA was detected in a wide range of domesticated animals of Europe, such as swine, small ruminants and horses in Denmark [9–11], and cattle in the UK [12]. Additionally, zoonotic transmission of *mecC*-MRSA from livestock to humans has been detected on several occasions [6, 13–15]. The first assumption was therefore that *mecC*-MRSA had a livestock reservoir, but the detection of *mecC*-MRSA in several species of European wildlife [4, 16–26] and in urban waste water [26] and river water [27] indicates otherwise [23]. The highest prevalence of human *mecC*-MRSA cases have been found in Denmark, accounting for 1–2% (30–50 cases annually) of all human MRSA cases, where they primarily cause skin and soft tissue infections [28]. In contrast to most MRSA isolates of human origin, *mecC*-MRSA does not seem to carry the φSa3 phage-encoded modulators of the human innate immune responses, including SAK (*sak*), CHIPS (*chp*), and SCIN (*scn*), which furthermore indicates a non–human origin [13].

### MRSA in European hedgehogs

The western European hedgehog (*Erinaceus europaeus*, hereafter referred to as “hedgehog”) is a small, spiny mammal which can be found throughout western Europe [29]. Hedgehogs are increasingly inhabiting areas with human activity, for instance gardens in residential areas and rural villages [30, 31], where garden owners are generously supplying food and water for hedgehogs [32]. The feeding of hedgehogs, combined with the tendency for hedgehogs to become habituated to human presence, makes the hedgehog one of the few wild mammals people are prone to come into physical contact with.

The first description of penicillin-resistant *Staphylococcus aureus* in hedgehogs was made in 1964, when Smith and Marples (1964)[33] isolated *S*. *aureus* in 40–71% of nasal, skin surface and paw swabs collected from 35 hedgehogs in New Zealand. A large proportion (79–92%) of these *S*. *aureus* isolates were resistant to penicillin. Smith (1965) [34] furthermore reported an *S*. *aureus* occurrence of 85% in 59 hedgehogs from New Zealand, with 86.3% being resistant to penicillin. Recently, Bengtsson et al. (2017)[16] detected *mecC*-MRSA in 64% of 55 hedgehogs from five counties in Sweden, of which most had died in care at wildlife rehabilitation centres. This detection rate of *mecC*-MRSA in hedgehogs seems extraordinarily high and indicates either dissemination of *mecC*-MRSA at the wildlife rehabilitation centres or, as also suggested by Bengtsson et al. 2017 [16], that hedgehogs might be an important natural reservoir for *mecC*-MRSA.

### The aim of the research

Previous studies on MRSA in wildlife have been limited by factors including small sample sizes, small geographical ranges, or by the use of samples from weak animals that have been in close contact with other wildlife species and humans during care at wildlife rehabilitation centres. Here we ameliorate these limitations by carrying out a large-scale nationwide study of wild hedgehogs in Denmark, aiming to: 1) determine the dissemination of MRSA in European hedgehogs throughout Denmark; 2) investigate determinants of MRSA carriage in hedgehogs; 3) determine the potential for zoonotic transmission of MRSA from hedgehogs to humans; and 4) characterise the detected MRSA on both a phenotypic and molecular level.

## Materials and methods

We established a nationwide citizen science project in Denmark to collect dead hedgehogs for a project aiming to understand more about hedgehog ecology. Volunteers were recruited via local and national media and a project website. Denmark consists of the large peninsula Jutland and several islands of differing sizes. The larger islands are connected by 0.75–17 km long bridges, which hedgehogs are unlikely to cross, isolating the local hedgehog populations. We strived to obtain samples from all of these regions. As a result of these efforts, citizens collected 697 hedgehogs throughout Denmark between May and December 2016. These animals were either found dead in traffic, from natural causes in the wild, or had died in care at wildlife rehabilitation centres.

In addition to collecting the dead animal, the volunteers were instructed to record the date and location of the find and deliver the hedgehog to the nearest of 26 collection stations, distributed nationally. All hedgehog carcasses were individually collected and sealed. Volunteers housing the collection stations emptied the collection bins daily, and stored the hedgehog carcasses in local freezers at -20°C before transportation to university laboratories, where they were thawed and necropsied. The necropsies took place from August 2016 to May 2018 and formed the basis for the present study.

### Sampling for detection of methicillin-resistant *S*. *aureus* in hedgehogs

A representative subsample of the collected hedgehogs was selected based on their geographical origin and tested for the presence of MRSA. To obtain a suitable sample for MRSA testing, a nasal swab was obtained from each individual just after thawing. A polyester tipped sterile applicator with a tip diameter of 1.98 mm (Puritan 25–1000 1PD) was used to swab the nares of the dead hedgehogs. The used applicator tip was subsequently placed in a sterilised 5.0 ml Eppendorf tube containing 700 μl of PBS and 300 μl of glycerol 50%, and was stored at -80°C.

### Investigation of MRSA transmission from hedgehogs to wildlife rehabilitators

To examine whether MRSA was present in humans with close contact to hedgehogs, 16 study participants working at wildlife rehabilitation centres under the Danish Animal Welfare Society were sampled from nose and tonsils in November 2017, and tested as described before by Angen et al.(2017)[35]. A short questionnaire about demographics was used to collect data from each participant (e.g., demographic characteristics, contact to wildlife, food animals, pets and medical history (Supporting information S1 Files). Data collection was approved by the Danish Data Protection Agency (protocol no. 2001-14-0021).

### Isolation and characterization of MRSA

The samples obtained from hedgehogs and wildlife rehabilitators were analysed for presence and characterization of MRSA at the National Reference Laboratory for Antimicrobial Resistance at Statens Serum Institut as described previously [10, 36]. PCR-based detection of *mecA*, *mecC*, *lukF-PV*, *scn*, and *spa* with subsequent *spa*-typing was performed by combining primers from two previously described multiplex PCR protocols [10, 36].

The PCR reactions were carried out in a final volume of 13μl containing 1 × Qiagen Multiplex PCR Master Mix (Qiagen, Germany), 2μM of each primer, and 1μl of bacterial DNA.

The following primer pairs were used: spa-1113f and spa-1514r [37]; MECA P4 and MECA P7 [38]; *mecA*_LGA251_MultiFP and *mecA*_LGA251_MultiRP [36], scn-F1 (5´-TACTTGCGGGAACTTTAGCAA-3´), scn-R1 (5`-AATTCATTAGCTAACTTTTCGTTTTGA-3´); PVL-FP and PVL-RP [39] FP2sau1 (5´-GAGAATGATTTTGTTTATAACCCTAG-3) and CC398r1[40] and 1μl DNA template (boiling lysate). The PCR consisted of a denaturation step (94°C, 15 min), 25 cycles of denaturation (94°C, 30 s), annealing (59°C, 1 min), and extension (72°C, 1 min), and a final elongation step (72°C, 10 min). PCR products were visualized on 2% E-Gels (Invitrogen, Grand Island, CA, USA). All *S*. *aureus* isolates were *spa*-typed as described previously by Harmsen et al. (2003)[37]. BURP cluster analysis of the *spa* types was performed in the Ridom StaphType software (Ridom GmbH, Germany) using default settings to deduce likely clonal complex (CC) types of *S*. *aureus* isolates. Simpson’s diversity index [41] was used to quantify *spa*-type diversity.

Antimicrobial susceptibility testing was performed by minimum inhibitory concentration (MIC) determination using a custom-made panel (DKSSP2, TREK Diagnostics), including 17 antimicrobials (penicillin, cefoxitin, ceftaroline, ceftobiprole, erythromycin, clindamycin, tetracycline, rifampicin, gentamicin, kanamycin, fusidic acid, sulfamethoxazole/trimethroprim, linezolid, mupirocin, vancomycin, daptomycin, norfloxacin). Interpretation of antimicrobial resistance was based on The European Committee on Antimicrobial Susceptibility Testing (EUCAST) breakpoints. For kanamycin and norfloxacin the breakpoints of Clinical and Laboratory Standards Institute (CLSI) were used. *S*. *aureus* ATCC 29213 was included as quality control of MIC determination.

### Habitat classification analysis

To assign the main habitat type to each hedgehog, all habitat types were extracted within a 500 m radius around where a hedgehog was found. This area is roughly equivalent to a large hedgehog home range[29]. The habitat classes were extracted using CORINE land cover data with a 100 x 100 m resolution (CLC 2012, Version 18.5.1). CORINE land cover data describes habitat types derived from satellite imagery divided into artificial surfaces: industry, agricultural areas, forest and semi-natural areas, wetlands and water bodies. For each area around which a hedgehog was found, habitat types were extracted in R using the raster package [42]. Afterwards, the habitat types were reclassified as “urban”, “rural” or “other” (Supporting information S1 Table). For further calculations, focus was on the percentages of urban versus rural, excluding other classes. The categorization of “urban” or “rural” was based on the highest percentagewise representation of the two categories for each individual hedgehog.

### Modelling determinants of MRSA carriage in hedgehogs

To investigate whether the detection of MRSA in a hedgehog was associated with sex, the region or habitat type in which it was found, or cause of death, we fitted generalized linear models (GLMs) in R [43] with binomial errors and a logit link function. The binary response variable was whether or not MRSA was detected and the explanatory variables included sex (female/male), region (a categorical variable), habitat (urban/rural) and cause of death (a 2-level categorical variable: in-care or natural/roadkill). We had small sample sizes from some regions and therefore we collapsed our region variables into four broader regions: 1) Jutland (north and south combined), 2) Lolland and Zealand together, 3) Funen, and 4) the islands of Bornholm, Møn, Samsø and Falster. We first fitted a maximal model including all explanatory variables and all two-way interactions between them (sex, cause of death, habitat, region). We then sequentially removed non-significant terms, interaction terms first and starting with the least significant, until we obtained a minimal adequate model where all remaining terms were significant [44]. We tested significance of term deletions with Chi-squared tests using the *dropterm* function from the MASS package [45].

### Ethics statement

According to the Danish Nature Agency an ethical approval to collect dead hedgehogs was not required for this research, regardless of their status as protected animals in the Danish legislation, because the hedgehogs used in the study had already died of natural causes either in the wild or in care at a hedgehog rehabilitation centre. The Danish Nature Agency furthermore informed us that a permission to collect dead hedgehogs from roads owned by the state was not necessary. We were hereafter encouraged to apply for permissions to collect dead hedgehogs from the roads owned by the 98 municipalities of Denmark, and we were authorised to do so after sending written inquiries to all 98 municipalities. We asked the volunteers to avoid collecting dead hedgehogs on private land and privately owned roads, as this would require a permission from the landowner. However, some of the hedgehogs were collected from private gardens owned by the volunteer collecting the dead hedgehog.

## Results

**Fig 1:**
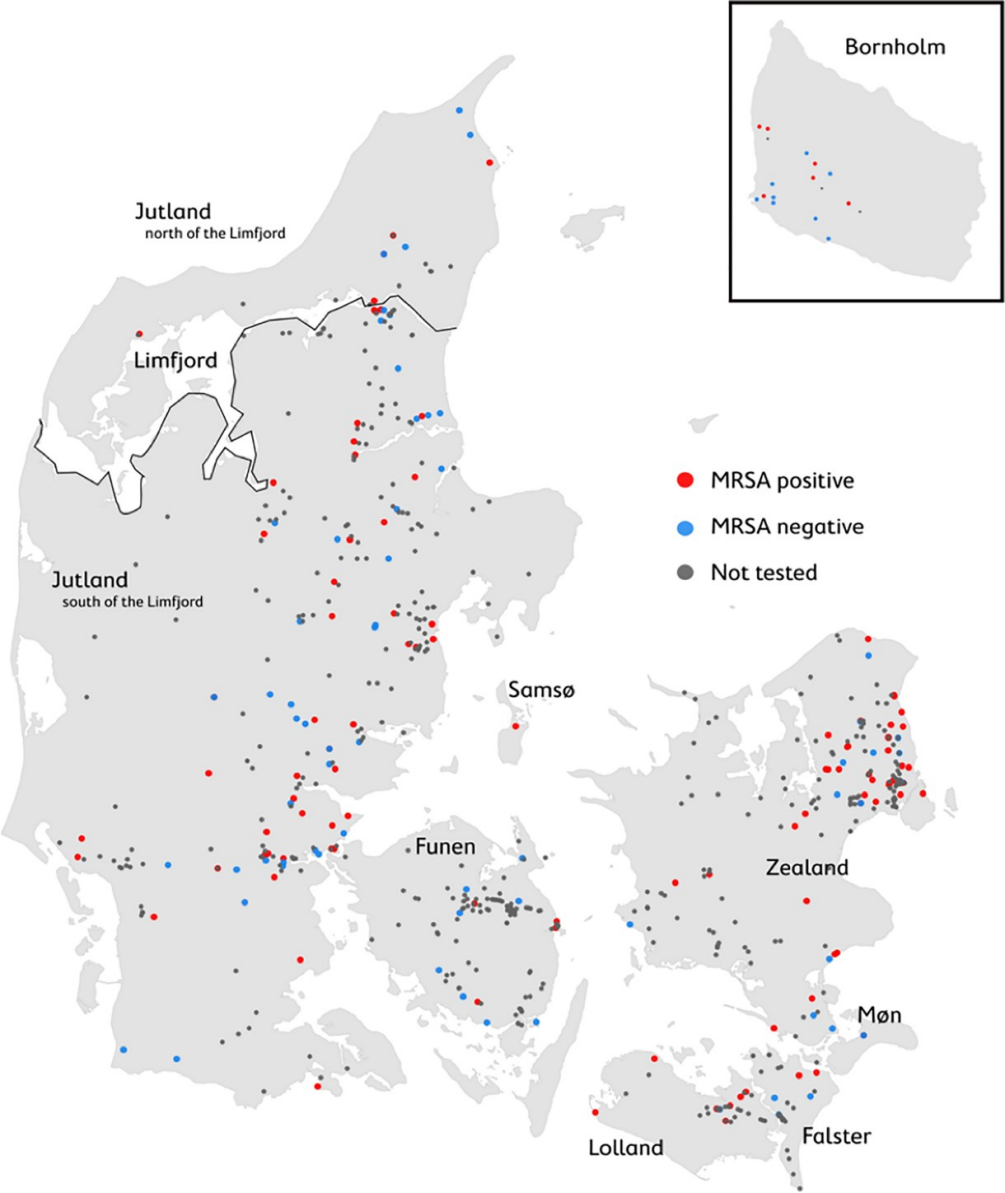
Map of *mecC*-MRSA distribution in hedgehogs. The distribution of the 697 collected, dead hedgehogs across Denmark. Each point represents an individual collected hedgehog. Grey points indicate that the individual was not MRSA tested, blue points indicate individuals that tested negative for MRSA, while red points indicate those that tested positive for *mecC*-MRSA.

Of the 697 hedgehogs collected throughout Denmark, we selected a subsample of 188 representing the different regions of Denmark (Table 1 and Fig 1). The selection of individuals for sampling was based on their condition (intact skull) and geographical locations to get the best representation of Denmark. The subsample consisted of 102 males, 56 females, and 30 individuals of unknown sex. Causes of death were 98 road-kills, 25 dying from natural causes in the wild, 16 dying in the wild from unknown causes and 49 dying in care. We found that 114 (61%) individuals carried *mec*C-MRSA and none carried *mecA*-MRSA. All MRSA isolates were susceptible to all tested antimicrobials except the β-lactams penicillin and cefoxitin. None of the isolates carried the genes encoding Panton-Valentine leukocidin (*lukF-PV*) or the modulator of the human innate immune response SCIN (*scn*).

**Table 1:** Occurrence of *mecC*-MRSA, CC-types and *spa*-types.

Region	No. tested/no. collected (% tested)	Occurrence of *mecC*-MRSA			*spa*-types		
		CC130	CC1943	Total	CC130	CC1943	D
Zealand	56/187 (30%)	46%	29%	75%	t843	t978, t3391, t8835	0.54
Funen	14/121 (12%)	29%	14%	43%	t528, t843	t3391	0.73
Jutland south of the Limfjord	79/277 (28%)	37%	19%	56%	t528, t843, t3256, t3570, t6220, t17133	t978, t2345, t3391	0.70
Jutland north of the Limfjord	8/20 (40%)	13%	38%	51%	t843	t978, t2345, t8835	1.00
Bornholm	14/18 (78%)	14%	29%	43%	t843	t8835	0.53
Møn	2/2 (100%)	50%	0%	50%	t843		NA
Lolland	10/36 (28%)	60%	20%	80%	t843, t1048	t978, t3391	0.64
Falster	4/30 (13%)	25%	25%	50%	t1048	t978	1.00
Samsø	1/1 (100%)	0%	100%	100%		t16868	NA
**Total**	**188/692 (27%)**	**37.2%**	**23.4%**	**60.6%**	**t528, t843, t1048, t3256, t3570, t6220, t17133**	**t2345, t3391, t8835, t16868, t978**	**0.69**

An overview of the results found when testing 188 dead hedgehogs for MRSA. The results are divided into areas of Denmark that are isolated from one another by the sea. Of the total 697 individuals collected, five were left out of the analyses per region due to lack of location data. Abbreviations: MRSA, methicillin-resistant *Staphylococcus aureus*; CC, clonal complex; D, Simpson’s index of diversity; NA, not applicable.

*mecC*-MRSA was present in animals from all areas and islands investigated (Table 1).

### Characterisation of the detected MRSA

**Fig 2:**
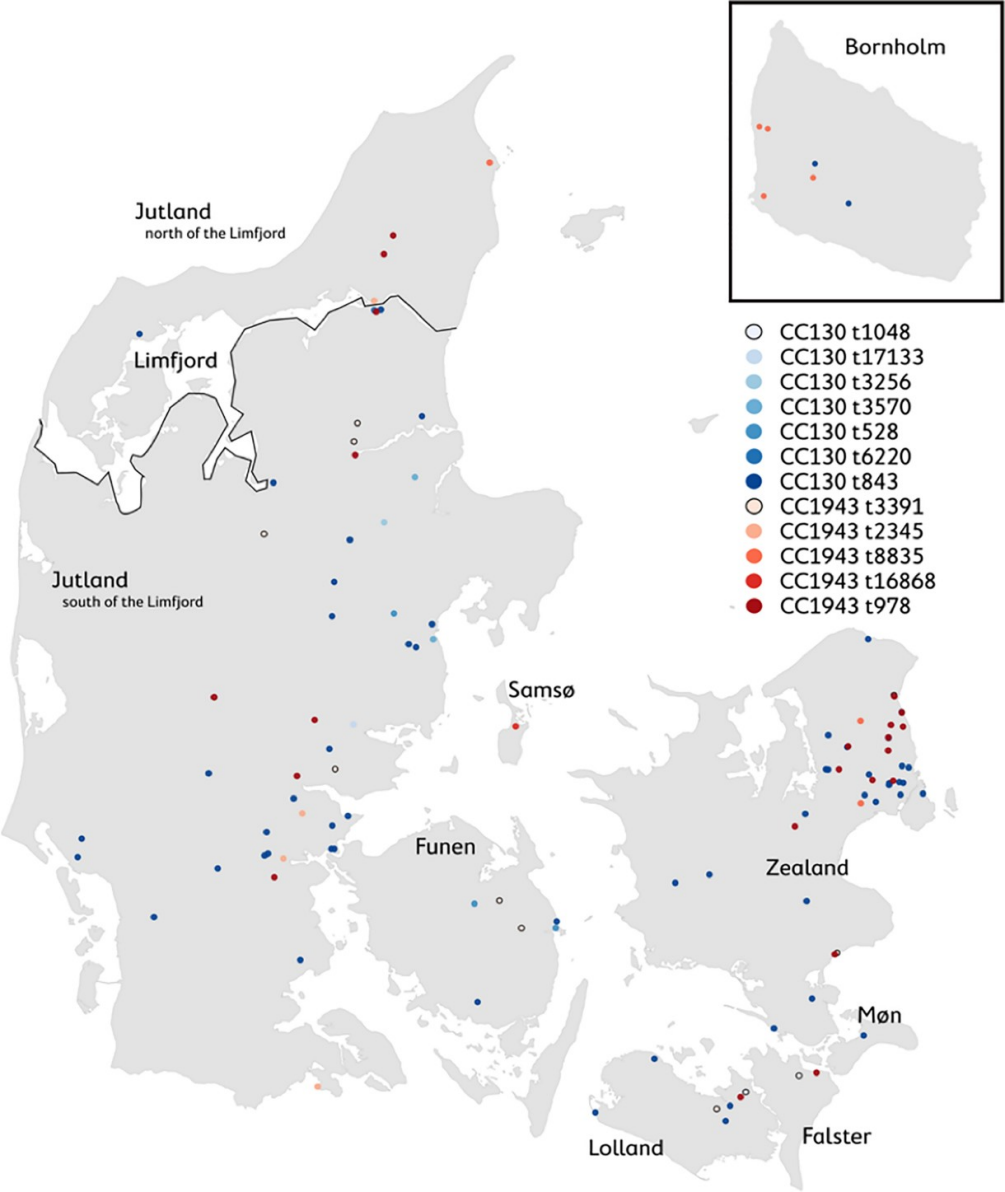
Map of CC-type and *spa*-type distribution. The distribution of the observed CC-types and *spa*-types of the 114 individuals carrying *mecC*-MRSA in Denmark.

The detected *mecC*-MRSA belonged to two genetic lineages CC130 (n = 70) and CC1943 (n = 44) and 12 different *spa*-types. The *spa*-types t528, t843, t1048, t3256, t3570, t6220, t17133 were associated with CC130, and the *spa*-types t978, t2345, t3391, t8835, t16868 were associated with CC1943. The occurrence of these CC- and *spa*-types appeared to vary geographically (Fig 2). CC1943 and CC130 were found in all areas, except for Møn (n = 2), which only had CC130. The most frequent *spa*-types were t843 (n = 59/114, 52%) followed by t978 (n = 22/114, 19%). Two new *spa*-types were described (t16868 on Samsø and t17133 in Jutland south of the Limfjord). The largest number of different *spa*-types was found in Jutland south of the Limfjord (n = 9), which also contributed with the largest sample size (n = 79). The *spa*-types t3256, t3570, t6220, t17133 were only found in this area of Denmark.

When comparing the distribution of CC-types between the two largest coherent areas of Denmark, Jutland south of the Limfjord (n = 79) and Zealand (n = 56) representing western and eastern Denmark, respectively, the distribution of CC130 and CC1943 was similar (66% and 34% in Jutland south of the Limfjord vs. 62% and 38% on Zealand). However, the *spa*-type diversity was higher in Jutland south of the Limfjord (Simpson’s D = 0.70) than on Zealand (0.54) (Table 1). The two areas shared three *spa-*types (t843, t978 and t3391).

A larger proportion of males (n = 67/102, 66%) than females (n = 31/56, 55%) were *mecC*-MRSA positive, but the difference was not found to be statistically significant (*p* = 0.67). Of the individuals with unknown sex 53% (n = 16/30) tested positive for *mecC*-MRSA.

No statistically significant differences were observed in the distribution and occurrence of MRSA based on cause of death: 57% of individuals that died in care (n = 28/49), 60% of individuals that died in the wild from natural causes (n = 15/25), 61% of road-killed individuals (n = 60/98) and 69% of individuals that died in the wild from unknown causes (n = 11/16).

### Habitat classification analysis

Our habitat analyses showed that 56% (n = 103) of the tested individuals resided in rural habitats and 44% (n = 81) in urban habitats, defined as industrial areas or residential areas, smaller or larger cities. Four individuals were excluded from the analyses because their percentagewise representations were equally distributed between “rural” and “urban”.

In total 56% (n = 58/103) of the individuals residing in rural areas and 65% (n = 53/81) living in urban areas carried *mecC*-MRSA (*p* = 0.99).

### Modelling determinants of MRSA carriage in hedgehogs

The minimal adequate model retained none of the explanatory variables, and the occurrence of MRSA was thus adequately explained by a model including only the intercept (0.454 ±0.174, expressed on the scale of the linear predictor (logit) used in the GLM). On the natural scale this represents a detection of 0.61 (95% CI = 0.53–0.69). Thus, the occurrence of MRSA in hedgehogs is not statistically significantly associated with sex or cause of death, nor does it vary significantly among regions, or between urban and rural habitats.

### Investigation of MRSA transmission from hedgehogs to wildlife rehabilitators

All hedgehog rehabilitators reported contact with hedgehogs, either on a daily (n = 3), weekly (n = 6), monthly (n = 3), or less than monthly (n = 4) basis. However, the nostril and throat samples from the hedgehog rehabilitators showed no growth of MRSA.

## Discussion

Our study confirms a high occurrence of *mecC*-MRSA isolates in Danish hedgehogs (61%), similar to the 64% presence of *mecC*-MRSA found in Sweden [16]. The larger sample size of hedgehogs as well as the inclusion of hedgehogs that died in the wild in our study provide additional evidence that hedgehogs are a natural reservoir of *mecC*-MRSA. Furthermore, our results indicate that the *mecC*-MRSA detected in our study is adapted to animals due to the lack of the *scn*-gene, which is a marker for human adapted *S*. *aureus* [46].

Other natural reservoirs than hedgehogs may exist, but it appears that European hedgehogs have a considerably higher occurrence of *mecC*-MRSA than other mammals. In comparison, Gomez et al. (2014)[18] detected *mecC*-MRSA in 2% of 101 faecal samples from six small mammal species in Spain, while 16.9% of nasal swabs obtained from 65 farmed red deer in Southern Spain were positive for *mecC*-MRSA [19].

Smith and Marples (1965)[47] and Bengtsson et al. (2017)[16] speculated that there could be a fitness advantage for *S*. *aureus* to become penicillin- and/or methicillin-resistant due to the presence of dermatophytes in hedgehogs producing penicillin-like substances [34]. This hypothesis seems reasonable, but further investigations are needed to provide any firm conclusions.

The subpopulations of Danish hedgehog are, to varying degrees, geographically isolated. This isolation is reflected by the differences in *spa*-types found in our study. As expected, the effects of this isolation were most pronounced on the smaller islands, e.g. Lolland and Falster (*spa*-type: t1048). The diversity of *spa*-types was higher in Jutland south of the Limfjord than on Zealand, which could indicate mixing with German hedgehog populations in Jutland south of the Limfjord. Interestingly, the three predominating *spa*-types reported from Southern Sweden (t843, t978, t3391) by Bengtsson et al. (2017)[16] also dominated in samples from Zealand.

The subsample of 188 individuals used in this study was stratified by region to ensure coverage across the entire country. However, we emphasize that only a few of the collected hedgehogs were representing the northwestern and southern parts of Jutland, due to difficulties with establishing collection stations there. Furthermore, our collection may not reflect the geographical hedgehog distribution in Denmark, since the collection of animals could have been influenced by the human population density and their commitment to the project.

We found a higher occurrence of *mecC*-MRSA in males than in females and, although this was not statistically significant. This is an interesting observation, because males likely have closer and more frequent contact with other individuals than females. This is both due to their promiscuous mating behaviour and due to the fights with other males, particularly during the mating season, where the males tend to physically fight off other males competing for the favour of the female [32]. In addition, home ranges of males are generally larger than those of the females[32], which would additionally mean that they are more likely to encounter more conspecifics than the females. Smith (1965)[34] also found a higher occurrence of *S*. *aureus* in males (69%) than females (40%).

Hedgehogs receiving care at wildlife rehabilitation centres may be more prone to MRSA acquisition due to their immunocompromised state, being housed closely together and receiving antibiotic treatments. However, we found no statistically significant difference in MRSA occurrence in hedgehogs dying at rehabilitation centres compared to other causes of death, indicating that hedgehogs can be carriers of *mecC*-MRSA regardless of their health status.

Increased population density leads to a greater disease transmission risk [48] and, therefore, one might expect a positive association between *mecC*-MRSA and hedgehog population density. Hedgehogs are progressively inhabiting human environments, for instance suburban residential gardens and villages in rural areas, as opposed to agricultural land [30, 31, 49]. Previous research in the UK [50] has furthermore indicated a greater decline in hedgehog densities in rural areas than suburban areas, so we expected a higher occurrence of MRSA positive individuals due to the higher population densities in urban and suburban habitats. However, we found no such difference.

We found no evidence for an association between MRSA presence and habitat type, region, cause of death, or sex. The high detection rate of MRSA in hedgehogs all over Denmark, strongly suggests that hedgehogs are natural reservoirs of *mecC*-MRSA.

A previous case of transmission of *mecC*-MRSA from hedgehogs to humans has been recorded in the National MRSA Register in Denmark, but none of the tested hedgehog rehabilitators tested positive for MRSA, indicating that zoonotic transmission of *mecC*-MRSA from hedgehogs to humans may occur only very rarely. It should however be emphasized that the human samples were collected in late November, which was approximately one month after the release of the last rehabilitated hedgehogs. Prolonged colonization with *mecC*–MRSA seems therefore not to have been established in these workers. Typing of *mec*C-MRSA from livestock indicates that zoonotic transmissions do occasionally occur, thus human cases may not get *mecC*-MRSA directly from hedgehogs but, rather, indirectly via livestock and pets. This speculation is supported by typing of the *mecC*-MRSA isolates from hedgehogs showing the same *spa*-types as reported before in both humans and livestock [6, 9, 11].

In conclusion, this nationwide study confirms a high occurrence of *mecC*-MRSA in Danish hedgehogs, which could serve as a natural reservoir for this type of MRSA. Other wild reservoirs may exist, but previous studies have only reported sporadic findings in other species (e.g. Gomez et al. (2014)[18]). Furthermore, this study found no sign of zoonotic transmission of *mecC*-MRSA from hedgehogs to wildlife rehabilitators caring for hedgehogs.

## Supporting information

S1 Files Questionnaire for the hedgehog rehabilitators. The questionnaire in an English and Danish version used as part of the research into the possible MRSA transmission from hedgehogs to wildlife rehabilitators.
(PDF)

S1 Table Reclassification of CORINE land cover habitat classes. CORINE land cover habitat classes found within hedgehog habitat, and how these were reclassified in either “urban”, “rural” or “other”.
(PDF)
