# Supplementary material for: European hedgehogs (Erinaceus europaeus) as a natural reservoir of methicillin-resistant Staphylococcus aureus carrying mecC in Denmark
Source: PLoS One. 2019 Sep 6;14(9):e0222031. doi: 10.1371/journal.pone.0222031 (PMC6730924; doi:10.1371/journal.pone.0222031)
Supplement: S1 Files — The questionnaire in an English and Danish version used as part of the research into the possible MRSA transmission from hedgehogs to wildlife rehabilitators. (PDF) [file pone.0222031.s001.pdf]

Code □□□□ **Date (dd/mm/yy) □□/□□/□□**

QUESTIONNAIRE

1. Personal data

1.1	Name:
1.2	Address:
1.3	Phone:
1.4	E-mail:
1.5	Gender: ❒ male ❒ female
1.6	Birthday (dd/mm/yy):
1.7	What is your occupation?

2. Contact to wildlife

2.1 Do you have regular contact with wildlife:

	Daily	Weekly	Monthly	Rarely	Direct contact (touched)	Only indirect contact
Hedgehogs						

3. Contact to farm animals or companion animals

3.3 Do you have regular contact with farm or companion animals:

	Daily	Weekly	Monthly	Rarely	Direct contact (touched)	Only indirect contact
Pigs						
Mink						
Dairy cows						
Veal calves						
Horses						
Sheep						
Goats						
Dogs						
Cats						

4. Other information		
4.1	Have you previously been diagnosed with MRSA?	❐ yes<br>❐ no<br>❐ don’t know
4.2	Have you within the previous 6 months lived together with a MRSA-positive person?	❐ yes<br>❐ no<br>❐ don’t know
4.3	Have you within the previous 6 months been admitted to a hospital or other healthcare facilities in a foreign country?	❐ yes<br>❐ no<br>❐ don’t know
4.4	Have you or a household member had weekly or more frequent contact to pigs within the previous 6 months?	❐ yes<br>❐ no<br>❐ don’t know
4.5	Have you within the previous 6 months lived of had daily contact to a nursing home or similar facilities or been admitted to a hospital unit during an MRSA outbreak?	❐ yes<br>❐ no<br>❐ don’t know
4.6	Have you within the previous 6 months been working in a hospital or other healthcare facility in a foreign country?	❐ yes<br>❐ no<br>❐ don’t know
4.7	Have you within the previous 6 months been working in a hospital unit, nursing home, or another healthcare facility during an MRSA outbreak?	❐ yes<br>❐ no<br>❐ don’t know
4.8	Have you within the previous 6 months been staying or working in a crowded place with poor hygiene facilities (e.g., asylum centers, homeless shelters, war zones, refugee camps or foreign orphanages)?	❐ yes<br>❐ no<br>❐ don’t know
4.9	Have you or one of your household members been working on a mink farm within the previous 6 months?	❐ yes<br>❐ no<br>❐ don’t know
4.10	Have you within the previous 6 months lived together with a person, who had been living in a foreign country within the previous 6 months (e.g., adoptees, au pairs, international travellers)?	❐ yes<br>❐ no<br>❐ don’t know
4.11	Have you within the previous 6 months had symptoms of skin infection while visiting a foreign country, in particular in relation to tattooing, piercing, sharing of equipment (e.g., diving) or imprisonment?	❐ yes<br>❐ no<br>❐ don’t know

Thanks for participating!

Kode ☐☐☐☐ **Dato (dd/mm/åå)** ☐☐/☐☐/☐☐

SPØRGESKEMA

**1. Personlige oplysninger**	
1.1	Navn:
1.2	Adresse:
1.3	Tlf. nr.:
1.4	E-mail:
1.5	Køn: ❒ mand ❒ kvinde
1.6	Fødselsdag (dd/mm/åå):
1.7	Hvad er din profession?

2. Kontakt til vilde dyr

2.1 Har du jævnligt kontakt til vilde dyr:

	Dagligt	Hver uge	Hver måned	Sjældent	Håndteret (rørt ved)	Kun indirekte kontakt
Pindsvin						

3. Kontakt til husdyr eller kæledyr

3.3 Har du jævnligt kontakt til husdyr eller kæledyr:

	Dagligt	Hver uge	Hver måned	Sjældent	Håndteret (rørt ved)	Kun indirekte kontakt
Svin						
Mink						
Malkekvæg						
Kødkvæg						
Heste						
Får						
Geder						
Hunde						
Katta						

4. Anden information		
4.1	Har du tidligere fået påvist MRSA?	❐ ja<br>❐ nej<br>❐ ved ikke
4.2	Har du inden for de sidste 6 måneder boet sammen med eller haft husstandslignende kontakt med MRSA-positiv person?	❐ ja<br>❐ nej<br>❐ ved ikke
4.3	Har du inden for de sidste 6 måneder modtaget behandling på hospital eller klinik i udlandet (uden for Norden)?	❐ ja<br>❐ nej<br>❐ ved ikke
4.4	Har du selv eller et husstandsmedlem haft ugentlig eller hyppigere kontakt til levende svin inden for de sidste 6 måneder?	❐ ja<br>❐ nej<br>❐ ved ikke
4.5	Har du inden for de sidste 6 måneder boet i eller haft dagligt ophold i plejeboliger eller lignende institutioner med MRSA-udbrud eller været indlagt på hospitalsafdeling?	❐ ja<br>❐ nej<br>❐ ved ikke
4.6	Har du inden for de sidste 6 måneder arbejdet på et udenlandsk hospital, plejebolig eller lignende institutioner uden for Norden?	❐ ja<br>❐ nej<br>❐ ved ikke
4.7	Har du inden for de sidste 6 måneder arbejdet på en afdeling på hospital, plejebolig eller lignende institutioner i Danmark og øvrige Norden, hvor der har været udbrud af MRSA på den pågældende afdeling?	❐ ja<br>❐ nej<br>❐ ved ikke
4.8	Har du inden for de sidste 6 måneder haft ophold eller arbejde under trange eller dårlige hygiejniske forhold (fx asylcenter, herberg for hjemløse, krigszoner, flygtningelejre eller på udenlandske børnehjem)?	❐ ja<br>❐ nej<br>❐ ved ikke
4.9	Har du inden for de sidste 6 måneder arbejdet på minkfarme eller er husstandsmedlem til person, der har arbejdet på minkfarme?	❐ ja<br>❐ nej<br>❐ ved ikke
4.10	Har du inden for de sidste 6 måneder haft husstandslignende kontakt med personer, der bor/har boet uden for Norden inden for de sidste 6 måneder (fx udenlandske adoptivbørn, au pair eller husstandsmedlemmer, der har været på længere udlandsrejse uden for Norden)?	❐ ja<br>❐ nej<br>❐ ved ikke
4.11	Har du inden for de sidste 6 måneder været i udlandet og har tegn/symptomer på stafylokokinfektion, specielt hvis personen har fået lavet tatovering eller piercing, delt udstyr (fx dykning) eller været i fængsel	❐ ja<br>❐ nej<br>❐ ved ikke

Tak for din deltagelse!
