# Supplementary material for: European hedgehogs (Erinaceus europaeus) as a natural reservoir of methicillin-resistant Staphylococcus aureus carrying mecC in Denmark
Source: PLoS One. 2019 Sep 6;14(9):e0222031. doi: 10.1371/journal.pone.0222031 (PMC6730924; doi:10.1371/journal.pone.0222031)
Supplement: S1 Table — CORINE land cover habitat classes found within hedgehog habitat, and how these were reclassified in either “urban”, “rural” or “other”. (PDF) [file pone.0222031.s002.pdf]

CORINE code	Label	Reclassed
111	Continuous urban fabric	urban
112	Discontinuous urban fabric	urban
121	Industrial or commercial units	urban
122	Road and rail networks and associated land	urban
123	Port areas	urban
124	Airports	urban
131	Mineral extraction sites	urban
132	Dump sites	urban
133	Construction sites	urban
141	Green urban areas	urban
142	Sport and leisure facilities	urban
211	Non-irrigated arable land	rural
222	Fruit trees and berry plantations	rural
231	Pastures	rural
242	Complex cultivation patterns	rural
243	Land principally occupied by agriculture	rural
311	Broad-leaved forest	other
312	Coniferous forest	other
313	Mixed forest	other
321	Natural grasslands	other
322	Moors and heathland	other
324	Transitional woodland-shrub	other
331	Beaches	other
411	Inland marshes	other
412	Peat bogs	other
421	Salt marshes	other
423	Intertidal flats	other
512	Water bodies	other

521	Coastal lagoons	other
523	Sea and ocean	other
